# Unraveling the role of cancer-associated fibroblasts in B cell lymphoma

**DOI:** 10.3389/fimmu.2024.1451791

**Published:** 2024-11-01

**Authors:** Prithviraj Mukherjee, Stephen M. Ansell, Patrizia Mondello

**Affiliations:** Division of Hematology, Mayo Clinic, Rochester, MN, United States

**Keywords:** fibroblast, tumor microenvironment, lymphoma, cancer associate fibroblasts (CAFs), immune response

## Abstract

Recent breakthroughs in research have sparked a paradigm shift in our understanding of cancer biology, uncovering the critical role of the crosstalk between tumor cells and the immune cells of the tumor microenvironment (TME) in malignant transformation. Fibroblasts have long been viewed as ancillary participants in cancer progression, often eclipsed by the prominence given to malignant cells. Novel investigations, however, have increasingly acknowledged the essential part played by the fibroblasts and their phenotypic doppelganger cancer-associated fibroblasts (CAFs) in fostering immunosuppression and promoting tumor progression. Here we review the cell-of-origin from which CAFs derive and their altered programs compared to their normal counterpart. We will also discuss the complex interplay between CAFs and the surrounding immune cells of the TME in the context of solid tumors and B cell lymphomas, with a focus on the “reprogrammable” role of CAFs in immunosuppression, immuno-activation and immuno-avoidance, and their implications on drug resistance. Finally, we will examine the existing and plausible therapeutic approaches targeting CAFs as a strategy to enhance treatment response.

## 1 Introduction

B cell lymphoma is a heterogenous group of tumors arising from the lymphocytes within the lymphoid structures (1). These diseases include a variety of subtypes with different clinical behavior, ranging from slow growing and indolent to fast and aggressive diseases, reflecting probably the nature of their progenitor cell. Considerable effort has been devoted to better understand the pathogenesis and progression of lymphoma. Seminal studies have uncovered the genetic alterations and dysregulated molecular pathways frequently involved in lymphomagenesis (2–4). Further investigation has applied clustering methods combining genomic and transcriptomic to classify molecular subtypes for personalized therapeutic approaches (5–9). However, a complex genetic background is insufficient to promote malignant transformation (10). Recently, the tumor microenvironment (TME) has demonstrated a fundamental role in enabling tumor development (11). The TME is composed by a plethora of highly plastic cells that can display anti-tumor or immune suppressive functions. The driver towards either immune fate is likely influenced by the tumor genetics and altered crosstalk between malignant and immune cells (12–15), however, recently the stroma cells have emerged for their central role in facilitating the formation of a pro-tumoral niche for the malignant cells (16). It is now well accepted that the stroma is more than just a scaffolding of the tissue and is involved in promoting and sustaining tumor development. The stroma also comprises the extracellular matrix (ECM) along with cytokine/chemokines and soluble factors, which are equally involved in the tumor-TME interaction.

**Figure 1:**
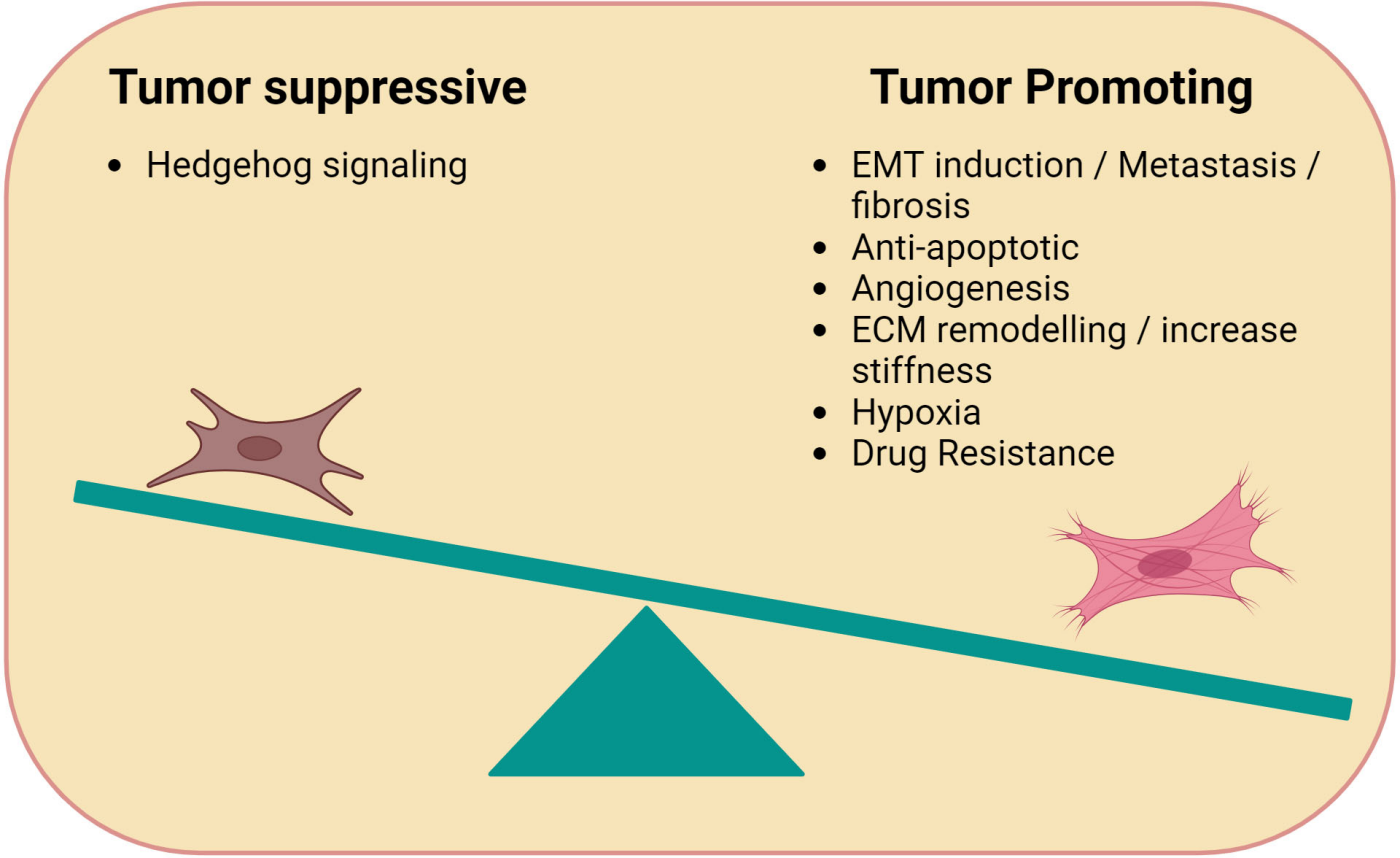
Immune-modulating functions of CAFs. Cancer Associated Fibroblasts (CAFs) can be tumor promoting or suppressing. Tumor promoting CAFs typically facilitate tumor growth through processes related to wound healing such as angiogenesis (e.g. VEGF), anti-apoptotic factors, ECM remodelling, and hypoxia. Tumor suppressive CAFs promote Hedgehog signaling.

Over the past decade numerous studies have demonstrated that many of the cellular and biochemical processes involved in the formation of the TME are closely related to the process of wound healing and inflammation (17, 18). One of the key players in the tumor cell-microenvironment interaction are the fibroblasts, also known as Cancer Associated Fibroblasts (CAFs) (19, 20). While healthy unaltered fibroblasts have the ability to secrete several growth factors and chemokines that are associated with wound healing to maintain structural integrity (21), CAFs use the same cytokines, soluble factors and degrading enzymes to remodel the TME (19, 22, 23). CAFs have a highly plastic, trans-differentiable phenotype that alters the biochemical and physical structure of the TME, along with altered effects on the pharmacokinetics within the TME (24, 25). For example, CAFs are able to secrete multiple pro-inflammatory cytokines such as interferon-γ (INF-γ), tumor necrosis factor (TNF-α), fibroblast growth factor (FGF) and vascular endothelial factor (VEGF) (26–28). Although CAFs display pro-tumorigenic effects leading to tumor progression, sometimes CAFs can also exhibit tumor suppressing qualities (**Figure 1**). One exception appears to be lymphoma where CAFs seems to mainly favor immune-escape (29). The malignant cells leverage these versatile properties of CAFs to promote tumor survival, proliferation, and disease progression. (30, 31). Extensive studies have been performed in solid tumors, while their characterization and function in lymphoma has not been adequately explored yet. In this review, we discuss the origin and multifarious nature of CAFs in lymphoma. Furthermore, we provide insights on ongoing investigation on therapeutic strategies targeting CAFs.

## 2 Fibroblasts and CAFs

### 2.1 Cell-of-origin of CAFs

Fibroblasts are non-hematopoietic cells of mesenchymal origin, with no specific surface biomarkers. They are usually identified as CD45^-^CD90^+^ cells, but their associated markers vary depending on the tissue in which they are located (32, 33). Their major function is the formation of ECM, through the production of collagen and fibronectin, which maintain structural integrity of the tissue and cell-cell interactions (34). Additionally, fibroblasts can secrete chemokines, cytokines and growth factors that are beneficial for wound healing and tissue repair (35). Of note, fibroblasts are not terminally differentiated and possess the capability to further differentiate from adipocytes, chondrocytes, osteoblasts and CAFs (36–40).

**Figure 2:**
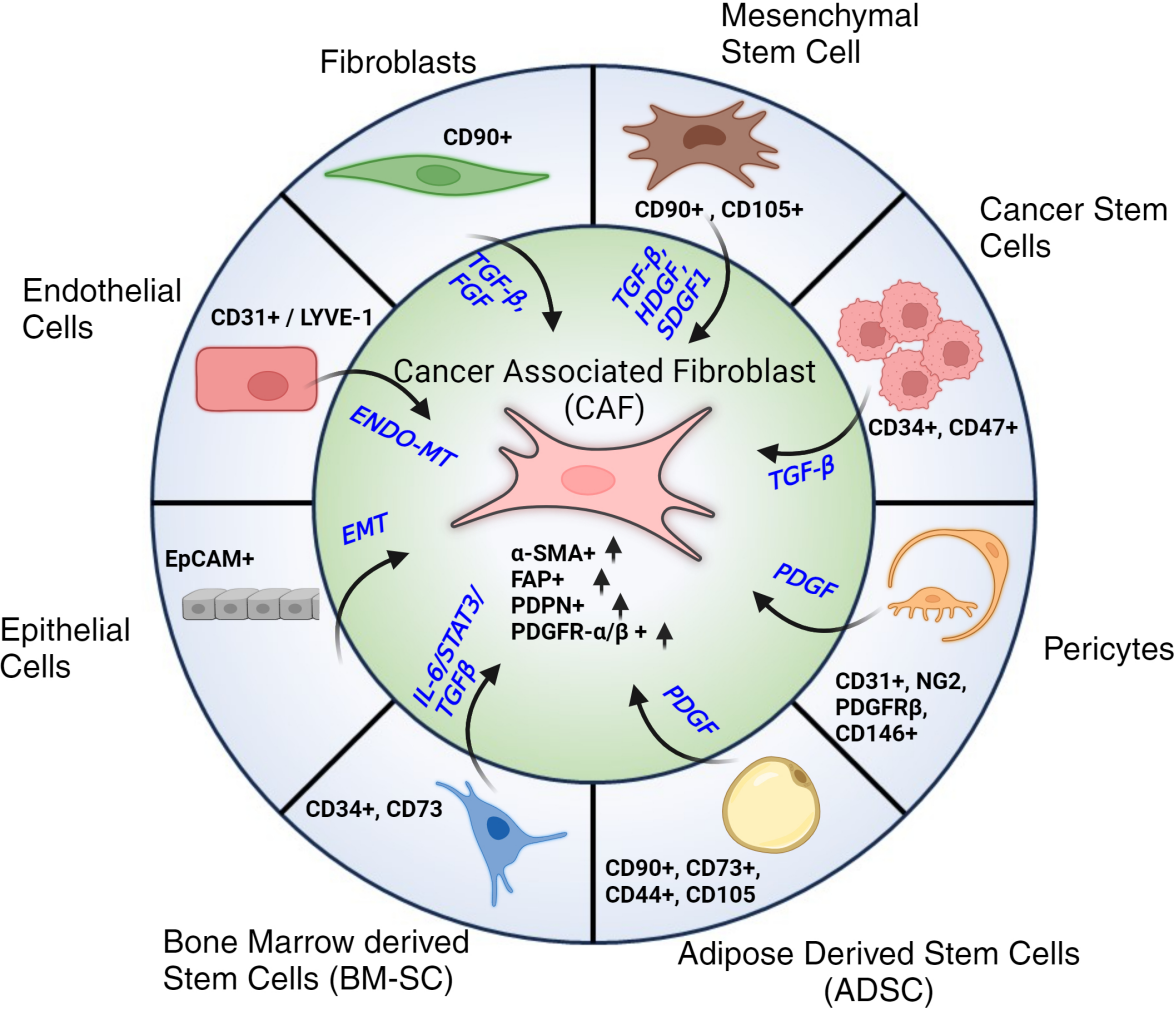
Cellular progenitors of CAFs. Cellular subtypes with their respective markers along with associated signaling pathways and signaling molecules that contribute to their trans-differentiation to Cancer Associated Fibroblasts (CAFs). Post differentiation CAFs are typically identified by their elevated expression of α-SMA, FAP, PDPN and PDGFR-α/β.

In contrast, our understanding of CAFs remains unclear (41). It is generally accepted that CAFs can have numerous cellular sources (42): CAFs can derive from activated fibroblasts, adipose tissue reservoirs, or myofibroblast-like cells (43, 44). CAFs can also derive from Endothelial to Mesenchymal transition (End-MT) and Epithelial to Mesenchymal Transition (EMT), driven by TGF-β signaling (24, 25, 30, 45). Other stromal cells (SCs) that can be the potential source of CAFs are pericytes, telocytes as well as cancer stem cells (CSCs). Each of these trans-differentiations is driven by a variety of factors that work in coordination as shown in **Figure 2**. The morphology of CAFs varies from stellate or cruciform to spindle shape (29, 36–40). Like fibroblasts, CAFs do not have specific markers. Typically they are identified as i) negative for hematopoietic markers (CD45^-^), ii) negative for epithelial/endothelial markers (CD31^-^), iii) high expression of α-Smooth Muscle Actin (α-SMA^+^), Fibroblast Activation Protein (FAP^+^), Podoplanin (PDPN^+^) and PDGFR α/β^+^ (22, 27, 28, 46, 47).

### 2.2 CAFs molecular classification

Single cell RNA-sequencing (scRNA-seq) has revealed that CAFs are a heterogenous population. Cords et al. identified 9 different clusters of CAF in breast cancer. These groups were classified as matrix CAF (mCAFs), inflammatory CAF (iCAFs), vascular CAF (vCAFs), tumor-like CAF (tCAFs), heat shock protein tumor like CAF (hsp-tCAFs), interferon-response CAF (ifnCAF), antigen presenting CAF (apCAF), reticular like CAF (rCAF) and dividing CAF (dCAF) (48), according to their unique gene expression profile. mCAFs were the most abundant, while rCAFs were the rarest. Notably, each cluster was defined by distinct gene pathway enrichment. For instance, mCAFs were identified by high expression of MMP11 and COL1A2, and upregulation of TGF-β and KRAS signaling, and pathways linked to myofibroblasts and EMT formation. These authors also subdivided CAFs into two broader groups: 1) the FAP^+^ group which included mCAF, iCAF, tCAF, hsp-tCAF, ifnCAF, apCAF, and dCAF, and 2) the FAP^-^ group which included vCAFs and rCAFs. This reclassification focused more on the functional heterogeneity rather than the genomic features of CAFs.

In lymphoma, the subtypes of CAFs have not been well defined as in breast cancer. One of the main reasons being the challenge associated with isolation of CAFs from lymph nodes (LN-CAFs) and bone marrow (BM-CAFs) due to the overlap between biomarkers of CAFs with other cell types. Single cell profiling of the stromal population has been performed through non-lineage isolation. For example, the Immunological Genome Project defined the gene expression of two populations of stromal cells and two populations of endothelial cells (EC) in LNs: i) PDPN^+^ CD31^-^ fibroblastic reticular cells (FRCs), ii) PDPN^-^ CD31^-^, double-negative cells (DNCs), iii) PDPN^+^ CD31^+^ lymphatic endothelial cells (LECs), and iv) PDPN^-^ CD31^+^ blood endothelial cells (BECs) (49). Rodda et al. identified nine clusters of non-endothelial origin cells based on specific anatomical and differential expression profiles of Ccl19^hi^ T-zone reticular cells (TRCs), Ccl19^lo^ TRCs (Ch25h^+^ cells in follicle-T-zone interface), Cxcl9^+^ TRCs (T-zone), marginal reticular cells (MRCs), perivascular (PvCs), CD34^+^ SCs, Inmt^+^ SCs, and follicular dendritic cells (FDCs) (50). These nine subsets were previously compiled into only two – FRC and DNC subtypes. One of the key highlights of this study was the identification of CXCL9^+^ TRCs and Nr4a1^+^ SCs as activated subtypes in several niches. Additionally, this study suggests that nodal SCs represent a transcriptionally distinct subtype which favors niche-restricted immune functions.

Recently, Abe et al. transcriptionally defined distinct LN-CAF subtypes. They identified 10 subclusters of BECs, 8 subclusters of LECs and 12 subclusters of non-endothelial SCs (NESC) in metastasis free lymph nodes (MFLNs). In lymphoma tissue, the same clusters showed substantial differences. For example, there was a higher proportion of BECs compared to MFLNs. Out of the 12 subclusters of NESCs, adventitial SCs (adv-SCs) proved to be a subtype of interest. Immunofluorescence (IF) staining of human follicular lymphoma (FL) LNs revealed localization of the Decorin^+^ fibroblasts, a strong marker for advSCs, SFRP4-SCs, SFRP2-SCs, TNF-SCs and C7-SCs that were found distributed throughout the adventitia, interfollicular regions (IFRs) and medulla. Moreover, this study also identified a CD70-CD27 focused interaction. CD70 is expressed in many adventitial SCs whereas CD27 is upregulated in FL B cells. Four out of five CD27^+^ FL samples showed obvious binding to CD70-Fc protein (51).

## 3 Functions of CAFs

### 3.1 Immunomodulatory role of CAFs

**Figure 3:**
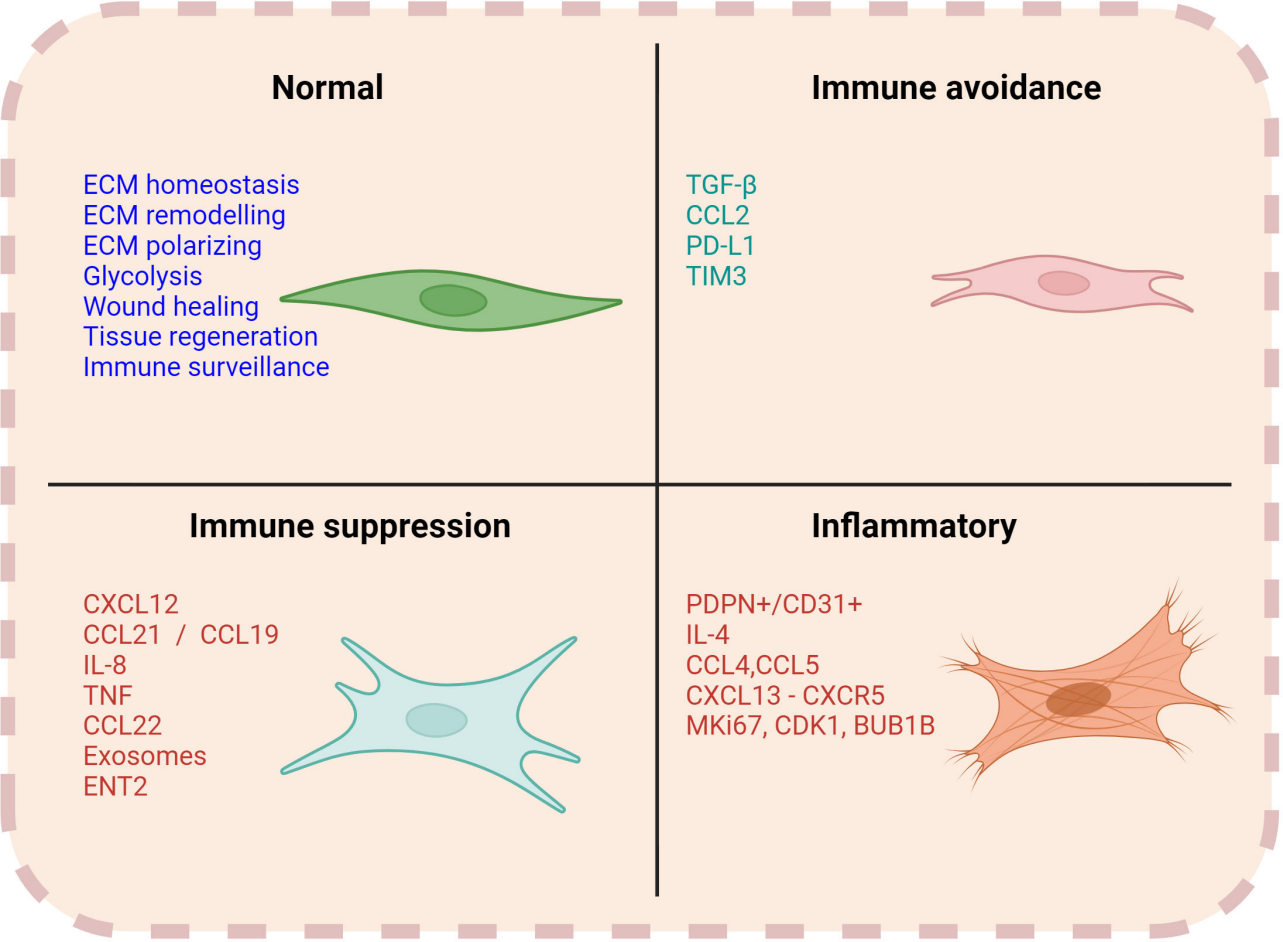
Functions of CAFs. Normal fibroblasts are engaged in several functions such as ECM remodelling, homeostasis, wound healing and immune surveillance. However, CAFs can have a multifaceted role in contributing to the immune regulation within the TME. Associated markers and soluble factors can vary with each of their respective functions.

The immunomodulatory effect of CAFs seems to be critically involved in favoring lymphoma cell growth and survival. However, this is a bidirectional process. Indeed, malignant B-cells can also promote the formation and expansion of CAFs (52–54). Besides altering their cell surface marker expression, malignant B cells can reprogram stromal cells to release cytokines, enzymes, and various soluble factors (e.g., exosomes and liposomal vesicles), which foster tumor progression and contribute to drug resistance (**Figure 3**). Mourcin et al. identified a subset of perivascular lymphoid stromal cells (LSCs) with high level of CD49a expression that display a pro-tumoral cytokines profile supporting FL B cells. In turn, FL B cells contribute to polarizing LSCs through TNF and TGF- activation pathway (16). Another subset of LSCs in FL overexpresses CXCL12 which triggers FL B cell expansion. Interestingly, polarization of CXCL12^hi^ stromal cells is promoted by IL4-producing T follicular helper cells, thus forming a pro-oncogenic IL4/CXCL12 loop (55). Similarly, in diffuse large B cell lymphoma (DLBCL) malignant B cells release lymphotoxin, which activates stromal cells and modulates their phenotype. In a reciprocal fashion, stromal cells release CLL19/CCL21 chemoattractant for malignant cells (56). Altogether these data support the existence of a circuit between fibroblasts and tumor cells that provides homing and pro-survival signals.

### 3.2 Immunosuppressive role of CAFs

A recent study by Apollonio et al. used primary samples from patients with DLBCL and IμHABcl6 mouse model, that recapitulates the genetics and biology of DLBCL (57) to investigate the functions of fibroblastic reticular cells (FRCs) in the TME (58). Transcriptional analysis of DLBCL-FRCs revealed upregulation of genes involved in proliferation (e.g., MKi67, CDK1, CDC20), metabolism (e.g., GYS2, STC1, KIF20), cellular adhesion (e.g., ITGA8, ICAM1, VCAM), and ECM (e.g., MMP9, MMP10, COL4A4). Gene set analysis showed enrichment for inflammatory response (e.g., IFN type -I and II), homing (e.g., ICAM) and antigen presentation signatures (e.g., MHC class I and II), with concordant loss of homeostatic signaling. High-definition imaging mass cytometry from 52 DLBCL samples revealed an expanded PDPN^+^CD31^-^ FRC population as compared to healthy tissues. These FRCs showed a myofibroblastic morphology with elevated expression of α-SMA. It is possible that FRCs are in part responsible for hindering migration of tumor infiltrating lymphocytes (TILs) and chimeric antigen receptor (CAR) T cells in DLBCL. Additionally, FRCs may be involved in limiting anti-tumor immune response due to their decreased expression in CCL21 and CCL19. Single cell profiling of CD45^-^ cells from LN of IμHABcl6 mice identified 7 clusters of FRCs subsets. Pathway analysis showed enriched fibroblast activation, ECM and IFN-response pathways. This is in line with the suppressive role of FRCs, which create a reticular meshwork limiting immune effector activity (57).

CAFs can also potentiate their immune-suppressive effect by cooperating with myeloid-derived suppressor cells (MDSCs). Studies in solid tumors have shown that MDSCs derived from monocytes that are recruited to the TME by the CCL2-CCR2 axis (59) and differentiate to MDSCs in response to IL-6 and GM-CSF, whose production has been attributed to CAFs (60, 61). This suggests that CAFs can recruit MDSCs in the TME, and together they inhibit T cell function and proliferation (62, 63).

### 3.3 Inflammatory role of CAFs

We and others have shown that the composition of the TME has prognostic implication in FL, DLBCL and other B cell lymphomas (64, 65). Although the research focus has been centered on T cells and macrophages, fibroblasts have recently attracted the attention of the scientific community. Thomazy et al. showed that the FRC meshwork in human FL has a higher expression of the enzyme/adhesion molecule transglutaminase (TG) compared to normal LNs (66). TG is normally expressed at low levels in the paracortex of primary follicles, however its expression was significantly upregulated in hyperplastic germinal centers of the neoplastic LNs. Interestingly, implementation of recombinant IL-4 (rIL-4) to the culture of FRCs induced 5 to 20-fold increase of TG (66), suggesting a role of IL4 in the modulation of FRCs. In a separate study, IL-4R expressing B cells interacted with CCL19^+^ FRCs to promote lymphoid formation (67, 68). Following the interaction with B cells, FRCs release CXCL-13 (67, 68), a well-known B-cell chemoattractant (69), further contributing to the lymphoid enlargement.

The expression of FDCs markers was found decreased in FL. Specifically, Chang et al. compared the expression of CD21, CD23, CD35, CXCL13, low-affinity nerve growth factor (LNGFR) and CAN.42 by immunohistochemistry (70). Out of 35 FL, there was partial to complete absence of FDC antigens in 31% of the FL cases. Loss of CD23 expression was the most common event followed by variable patterns of LNGFR and CAN.42. In contrast, CXCL13 expression pattern was similar in both neoplastic and reactive follicles, but not in extrafollicular zones. Notably, the staining for CD21, CD23, CD35, CXCL13 was negative in extrafollicular stroma, suggesting that mature FDCs do not differentiate or migrate to intrafollicular zones in FL.

Most recently, Radtke et al. reported a detailed molecular and spatial atlas of FL TME (71). Their comprehensive multiomics approach, including spatial imaging, bulk and scRNA-seq of normal and malignant LNs from untreated FL patients, revealed several distinguishing characteristics in high-risk FL patients. Neoplastic follicles from high-risk early relapsed patients were smaller, irregularly shaped and separated by a greater distance than those from low-risk patients, which had a back-to-back follicle distribution and a smaller distance of separation. Histological analysis showed an increase in DC-SIGN^+^ myeloid cells and desmin^+^ FRCs (CD21^+^ CD23^+^ CD35^+^) in high-risk patients. Additionally, spatial imaging analysis revealed that these FRCs were positive for CD49a and CXCL13. Gene set analysis of patients who relapsed early compared to those with late relapse revealed an enrichment for BCR (B-cell Receptor) signaling pathways, ECM remodeling, fibrosis, MMPs, collagen deposition and glucose metabolism. These findings uncovered the occurrence of stromal desmoplasia, and abnormal follicular growth patterns in FL patients with poor prognosis. Bulk RNA seq from early relapse LNs identified high expression of TGF-β, CCL4, CCL5 and CXCL13 genes, which are linked to cell recruitment within the TME. Paired scRNA-seq confirmed upregulation of CXCL13-CXCR5 gene set, suggesting lymphocyte infiltration within the TME. In line with prior observations (72, 73), IRF4^+^ B cells were found in close association with the DC-SIGN^+^ myeloid cells, T cells and vimentin^+^ cells. Collectively, these data highlight the importance of stromal FDC and FRC phenotype cells in stromal modulation and suggest their potential prognostic role in newly diagnosed FL.

### 3.4 Role of CAFs in immuno-avoidance/evasion

Malignant cells achieve immune evasion by several strategies including 1) restriction of antigen presentation through downregulation or loss of MHC I-II, co-stimulatory immune molecules, and adhesion molecules (CD54/I-CAM), 2) reducing immunogenicity and 3) inducing T cell exhaustion (74–77). In this regard, the stromal cells assist malignant cells in further promoting immune escape. CAFs are responsible for forming an immune barrier by virtue of their ability to secrete both myeloid and lymphoid like chemokines, TGF-β being one of the most important as mentioned previously (78). In mice, BM-CAFs have shown to attract tumor associated macrophages (TAMs), by secreting CCL2 which binds to CCR2 on monocytes (79, 80). Once recruited within the TME, BM-CAFs are known to drive M2-like TAM differentiation through secretion of GM-CSF, M-CSF and IL-6 (78, 81). M2-like TAMs promote immune evasion by blocking anti-tumor Th1 cells (82). Additionally, the GM-CSF released in the TME can support immune-evasion by promoting PD-L1 expression on extranodal natural killer (NK) cells/cytotoxic T cells (83). Lastly, lymphoma neovascularization has shown to act as a functional barrier that facilitates immune tolerance. Microdissection of the endothelium identified increase expression of TIM-3, which was correlated to a poor prognosis (84, 85).

## 4 Therapeutic strategies for CAFs

In the context of immunotherapy CAFs have gained attention for their immunomodulatory functions. CAFs can influence immune cell recruitment, activation, and function within the TME. Additionally, CAFs increase drug resistance through multiple mechanisms including dysregulation of glycolysis and secretion of soluble molecules/exosomes (22). Therefore, targeting CAFs in combination with other immunotherapy has been an ongoing effort and has emerged as a promising strategy to enhance clinical responses.

CAFs are known for their contribution in cancer progression by supporting the growth of cancer stem-like cells through paracrine secretion of growth factors (86–89). Similarly, the close association of CAFs to malignant cells may lead to increased tumor growth and proliferation and may display a protective role from immune surveillance (90). Typically, chemotherapy induces cytotoxicity along with recruitment of cytotoxic immune cells (91–94). CAFs activity in the TME can render these therapies ineffective by blocking immune infiltration. CAFs can also assist in immune escape of malignant cells from chemotherapy or radiotherapy by their close association to CSCs. CSCs represent a quiescent or slow-growing cell population that lays dormant within the tumor, making it elusive to chemotherapy treatments (95, 96).

In lymphoma, LN-CAFs can secrete increased pyruvate levels, which help in the reduction of reactive oxygen species (ROS), augment cell-cell interaction and the citric cycle which is important for malignant cell survival (97). Moreover, LN-CAFs in lymphoma can also secrete exosomes containing the pyrimidine transporter protein ENT2 (98), which contains miR-4717, a microRNA thought to be responsible for upregulation of PD-1 expression (99). In DLBCL, CAFs were found to express PD-L1, thereby reducing the cytotoxic activity of CD8^+^ T cells (100–102). In contrast, in adult T-cell leukemia/lymphoma (ATLL), CAFs promote CD4^+^ T cell proliferation via FGF7-FGF1 and PDGFA-PDGFRA/B signaling (102). These CAFs were classified into two subgroups - EGR_high_ and EGR _low_ of which EGR_high_ seemed to promote NK and T-cell efficacy, suggesting a potential benefit of CAF-directed therapy in ATLL.

Although the underlying mechanisms of CAFs formation remain enigmatic, their altered pathways have been explored in hematological malignancies as promising therapeutic targets. Therapy directed against CAFs can be categorized in i) antibodies, ii) secreted factors, and iii) cytokines/chemokines. The effect of TGF-β signaling in CAFs formation was explored by two groups. Pan et al. reported that TGF-β promotes a phenotypic differentiation of bone marrow mesenchymal stem cells (BM-MSCs) into CAF-like cells (103). Accordingly, inhibition of TGF-β lowered BM-CAFs activation (104, 105). BM-CAFs differentiation from BM-MSCs was also shown to be dependent on SDF-CXCR4 pathway in multiple myeloma (MM) (106, 107), while CXCR4 inhibitors (e.g., BL-8040) resulted in CAFs removal with promising clinical responses (108, 109). LOX/LOX2 signaling, related to ECM and collagen formation in the TME (110), is also upregulated in hematological malignancies (111). For example, LOX2 was found to be closely related to the process of myofibrosis/CAF formation in myeloproliferative neoplasms (MPN) (112). Thus, LOX and LOX2 inhibitors may represent promising therapeutic strategies. Additionally, targeting the CD70-CD27 axis has shown promise in FL as blocking CD27 decreased the number of malignant B cells (51).

Finally, CAFs seem sensitive to anti-angiogenic drugs (e.g., bevacizumab, sorafenib, sunitinib), as they secrete a number of angiogenic growth factors such as VEGF (113). Crawford et al. show that CAFs from anti-VEGF resistant TIB6 murine lymphoma demonstrated drug response by revascularization of PDGF-C in both *in-vitro* and *in-vivo* experiments (114).

## 5 Conclusion

In conclusion, CAFs are a multi-faceted subset of cells within the lymphoma TME that can have varied sources of origin like fibroblasts, MSCs and EMT. The interaction between CAFs and immune cells in lymphoma is complex and multifactorial and may play a critical role in tumor progression and/or relapse and treatment resistance. Understanding the mechanisms underlying the action of CAFs and their interactions with the surrounding TME is crucial for developing more effective therapies to enhance anti-tumor immune responses in lymphoma.
